# A prospective diagnostic study investigating urinary biomarkers of AKI in major abdominal surgery (the AKI-biomas study)

**DOI:** 10.1186/s13054-025-05510-8

**Published:** 2025-07-01

**Authors:** Rishabh Singh, William Maclean, Toolika Singh, Paul Mackenzie, Timothy Rockall, Lui G. Forni

**Affiliations:** 1 Department of Critical Care Medicine, Royal Surrey Hospital NHS Foundation Trust, Guildford, GU2 7XX UK; 2 Department of General Surgery, Royal Surrey Hospital Foundation Trust, Guildford, GU2 7XX UK; 3 School of Medicine, Kate Granger Building, University of Surrey, Guildford, UK; 4 Intensive Care Unit, Royal Surrey Hospital NHS Foundation Trust, Guildford GU2 7XX UK, UK

**Keywords:** AKI (Acute kidney Injury), NGAL (Neutrophil gelatinase-associated lipocalin), KIM-1 (Kidney injury molecule-1), DKK-3 (Dicckopf-related protein 3), IGFBP-7 (insulin-like growth factor binding protein-7) and TIMP-2 (tissue inhibitor of metaloproteinase-2)

## Abstract

**Background:**

Post-operative acute kidney injury (AKI) is associated with increased morbidity and mortality with evidence suggesting that early identification using biomarkers of AKI may impact prognosis. Most studies in surgical patients has focussed on cardiac, vascular and transplant surgery cohorts. Evidence on the utility of biomarkers in major abdominal surgery is sparse.

**Methods:**

This was a prospective observational single centre diagnostic study conducted on 488 patients undergoing major abdominal surgery. Urine was collected four hours post-surgery. The biomarkers for AKI NGAL, KIM-1, DKK-3 and IGFBP-7*TIMP-2 were measured and diagnostic performance assessed utilising Receiver Operating Characteristic (ROC) curve analysis to predict the development of post operative AKI using serum creatinine and urine output criteria.

**Results:**

242 participants developed AKI by urine output criteria (49.5%) and 43 by serum creatinine criteria (8.8%). The area under the receiver operating characteristic curve values for stage 1 AKI as determined by serum creatinine criteria for NGAL was 0.741 (95%CI 0.699–0.770, *p* < 0.001) and 0.871 (95%CI 0.838-0.899, *p* < 0.001) for stage 2. AUC values for IGFBP-7*TIMP-2 for stage 1 were 0.655 (95% CI 0.611–0.697, p0.003) and stage 2 0.803 (95%CI 0.764–0.837 p0.002). The AUC for KIM-1 was statistically significant for stage 1 (0.68, 95%CI 0.637–0.722) but not for stage 2. No AUC values for DKK-3 were statistically significant. Biomarkers performed poorly for prediction of AKI by urine output criteria.

**Conclusions:**

In this large prospective study of a clinical cohort of 488 patients undergoing major abdominal surgery AKI rates are dependent on the criteria used with 49.5% of patients developed AKI by urine output criteria, compared to only 8.8% by serum creatinine. NGAL and IGFBP-7*TIMP-2 showed reasonable diagnostic performance when diagnosing AKI by serum creatinine criteria, with NGAL returning the highest AUC values.

**Supplementary Information:**

The online version contains supplementary material available at 10.1186/s13054-025-05510-8.

## Background

The development of acute kidney injury (AKI) in the post operative period is associated with an increased incidence of mortality and significant co-morbidity including an increased risk of developing chronic kidney disease (CKD) [1, 2]. However, the prevalence of AKI in general surgical patients is dependent on both patient factors as well as the type of surgery, hence the wide range of reported incidence rates [3–7]. For example, liver transplantation and cardiac surgery have reported AKI rates of more than 50% whereas in those undergoing neurosurgery observed rates are much lower [8–10]. Currently, AKI is defined through observed changes in both serum creatinine (SCr) and weight adjusted urine output (UO) as described by the Kidney Disease: Improving Global Outcomes (KDIGO) criteria [11]. However, this approach has significant limitations in surgical populations given that oliguria may be affected not only by volume resuscitation and diuretic usage but may also reflect the normal physiological response to surgical stress [12, 13]. Similarly, SCr concentrations are affected by gender, nutritional status, production rate and to a lesser degree, volume resuscitation, factors likely compounded in the critically ill where small alterations in SCr levels, even reductions, may portend negative outcomes [14, 15]. Importantly, initial rises in SCr may materialise only once glomerular filtration has fallen significantly, which may take up to 48 h post insult [16, 17]. 

At present there is no treatment for AKI, therefore interest has focussed on early identification including biomarkers which may allow timely intervention to minimise progression, potentially translating into improved outcomes. If biomarkers can identify renal stress or damage as early as the immediate post-operative period, then one may instigate prompt treatment decisions prior to those triggered by changes in SCr or UO. To-date, most biomarker studies in surgical patients have focussed on cardiac, transplant and vascular procedures. Indeed, biomarker use is encouraged in cardiac Enhanced Recovery (ERAS) Programmes [18]. However, data from these patient groups may not translate directly to patients undergoing major abdominal surgery. Studies assessing biomarker performance in major gastrointestinal or gynaecological operations are uncommon and when included they tend to be pooled within larger cohorts of cardiac, transplant and vascular surgery [19–25]. In this study we have concentrated on patients undergoing major abdominal surgery with the aim to assess the diagnostic performance of several available AKI biomarkers in this patient cohort.

## Methods

### Study aim, design and setting

This was a single centre prospective, observational diagnostic study assessing urinary biomarkers for the prediction of post-operative AKI in major abdominal surgery, defined as operations where the peritoneal cavity is entered and mortality risk is greater than 1%.^25^ Ethical approval was obtained from the London Queen’s Square Research Ethics Committee and the Health Research Authority (ref: 18/LO/0601) and this study was registered at clinicaltrials.gov (ref: NCT04582747; Registered 4/7/2018). Written consent was obtained from all participants.

The study follows the Strengthening of Reporting in Observational Studies in Epidemiology (STROBE) and Standards for Reporting of Diagnostic Accuracy (STARD) criteria [26, 27]. 

### Study population

Data was collected on patients 18 and older undergoing major abdominal surgery either routinely or emergently between October 2018 and January 2020. Only patients requiring a urinary catheter were included to facilitate accurate urine data collection. Patients were admitted to the general surgery ward or intensive care depending on clinical need. Individuals on renal replacement therapy, those without pre-operative SCr measurements and those with AKI at recruitment were excluded. The sample size calculation assumed an expected prevalence of AKI of 10% based on prior literature for AKI diagnosed by SCr, a desire for a 95% confidence interval (CI) around the diagnostic performance estimate, and a precision of 10%. The target recruitment was 471 with 500 samples collected to factor in unplanned exclusions [28]. Non-emergent surgery was performed according to speciality specific ERAS guidelines [29–33]. 

### Biomarker collection and analysis

Urine samples were obtained four hours post abdominal closure given previous studies have determined that this period enables clinically useful predictions regarding AKI can be made [23]. Samples were immediately centrifuged at 15–30 °C for ten minutes at 1000 g then stored at < −70 °C [22]. Supernatant samples were transported on dry ice with temperature monitoring for biomarker testing in external laboratories. Biomarkers studied included Neutrophil gelatinase-associated lipocalin (NGAL), Kidney injury molecule-1 (KIM-1), Dicckopf-related protein 3 (DKK-3) and the product of insulin-like growth factor binding protein-7 (IGFBP-7) and tissue inhibitor of metaloproteinase-2 (TIMP-2). These biomarkers were selected based on their differing pathophysiological targets—NGAL and KIM-1 as damage markers, DKK-3 as a tubular stress marker, and IGFBP-7*TIMP-2 as stress response markers—allowing us to compare performance across biological pathways.

The Nephrocheck^TM^assay. Testing for IGFBP-7 and TIMP-2 was conducted by Ortho Clinical Diagnostics using the Vitros Nephrocheck™ (NC) Immunoassay. Testing for DKK-3, NGAL and KIM-1 was conducted by Affinity Biomarker Laboratories, a United Kingdom Accreditation Service certified laboratory. Testing for NGAL and KIM-1 utilised the R&D Systems Quantikine™ Immunoassay, DKK-3 the Sigma-Aldrich/Millipore^R^ enzyme-linked Immunosorbent assay. Laboratory technicians were blinded to trial data. Results were made available for analysis at the end of study recruitment. There were no adverse events resulting from urine collection.

### Study outcomes

AKI was defined by the KDIGO criteria, utilising both UO (AKI-UO) and SCr (AKI-SCr) and included patients not meeting the classical AKI criteria but with a positive biomarker result, described as Stage 1S [34]. In patients meeting both SCr and UO criteria patients were classified under AKI-SCr for subgroup analysis purposes. When determining AKI stage severity, we used the higher KDIGO stage between SCr and UO. SCr measurements were required at least once within three days post-operatively with UO monitored until three days post-operatively, or until removal of the urinary catheter if earlier. Adverse outcomes were classified as an increased length of hospital or intensive care stay, 30 and 90-day mortality, and the development of Clavien-Dindo stage 3 or higher post-operative complications during the hospital admission [35]. Biomarkers were also assessed for ability to predict long term changes in renal function. This was assessed by classifying the participants change in their ‘CKD’ stage from the pre-operative status, determined by the measured eGFR at follow up (at least 6 months from their operation) as per the KDIGO criteria. The ability of biomarkers to predict mortality within 1 year was also assessed. Biomarker performance was assessed utilising AUROC analysis and calculating the relative risk of an increase in CKD stage or 1-year mortality if biomarker positive. Relative risk of 1-year mortality and increase in CKD stage was also analysed for those who did not develop AKI by any criteria, and those who did by either AKIUO or AKISCr.

### Statistical analysis

Statistical analysis was performed using MedCalc^®^ version 19.4.1. Due to the skewed nature of the data median values are provided. Categorical data is provided as frequency counts and percentages. Biomarker performance was assessed for the prediction of AKI-UO and AKI-SCr utilising Receiver Operating Characteristic (ROC) curve analysis [36]. Significance and 95% confidence intervals (CI) of the area under receiver operator characteristic curve (AUC) are provided and identification of the optimum cut-off employed the Youden Index (YI) [37, 38]. ROC analysis was also conducted to assess predictive ability for adverse outcomes. The YI from biomarker ROC analysis for AKI-SCr stage 1 was used to determine ‘biomarker positivity’. Odds of developing AKI and adverse outcomes were compared with the Mann-Whitney test. K-fold cross validation was performed with a K-value of five, a method shown to be an effective means of internal validation [39, 40]. A mean ‘test’ AUC was calculated and compared to the AUC achieved from the overall study. Characteristics of each test and train group were also compared, utilising Mann-Whitney and Chi squared tests as appropriate.

## Results

**Baseline characteristics**: A total of 596 patients were recruited with 488 (median age (IQR): 65 (54–73) years) included in the final analyses, and most patients were women (53.9%]). 146 (29.9%) underwent colorectal surgery, 209 (42.8%) hepatobiliary, 75 (15.4%) gynae-oncological and 40 (8.2%) oesophagogastric surgery (Table 1). Follow up renal function was obtained for a median (IQR) of 398 (366–490) days postoperatively. Patients in the emergency group were highlighted to the research team if normal pre-operative SCr levels were obtained. Median time of urine collection was 241 min post procedure (IQR – 230–250 min) and 93.9% of participants had SCr measurements on the first post-operative day, 86.5% the second post-operative day and 73.7% the third post-operative day. 22.1% had urinary catheters removed on the first post-operative day and 27.8% the second post-operative day. 50.1% retained a catheter on the third post-operative day and beyond. 437 (89.5%) patients underwent elective surgery, 51(10.5%) emergency surgery.

**Table 1:** Baseline Characteristics of Whole Cohort

	**Total (%)**	**AKI-SCr**	**AKI-UO**	**No AKI**
Female	263 (53.9)	15 (34.9)	130 (53.7)	134 (54.7)
Male	225 (46.1)	28 (65.1)	112 (46.3)	111 (45.3)
ASA 1	11 (2.3)	0 (0)	3 (1.2)	7 (2.9)
ASA 2	362 (74.2)	20 (46.5)	170 (70.3)	194 (79.2)
ASA 3	105 (21.5)	20 (46.5)	66 (27.3)	39 (15.9)
ASA 4	10 (2)	3 (7)	3 (1.2)	5 (2)
CKD	87 (17.8)	14 (32.5)	47 (19.4)	39 (15.9)
T1DM	10 (2.1)	3 (7)	4 (1.7)	6 (2.4)
T2DM	51 (10.5)	7 (16.3)	28 (11.6)	22 (9)
Hypertension	166 (34)	22 (51.2)	88 (36.4)	74 (30.2)
ACE Inhibitor	61 (12.5)	6 (14)	34 (14)	25 (10.2)
Angiotensin receptor blocker	36 (7.4)	6 (14)	17 (7)	15 (6.1)
Elective Surgery	437 (89.5)	36 (83.7)	212 (87.6)	224 (91.4)
Emergency Surgery	51 (10.5)	7 (16.3)	30 (12.4)	21 (8.6)

**Fig. 1:**
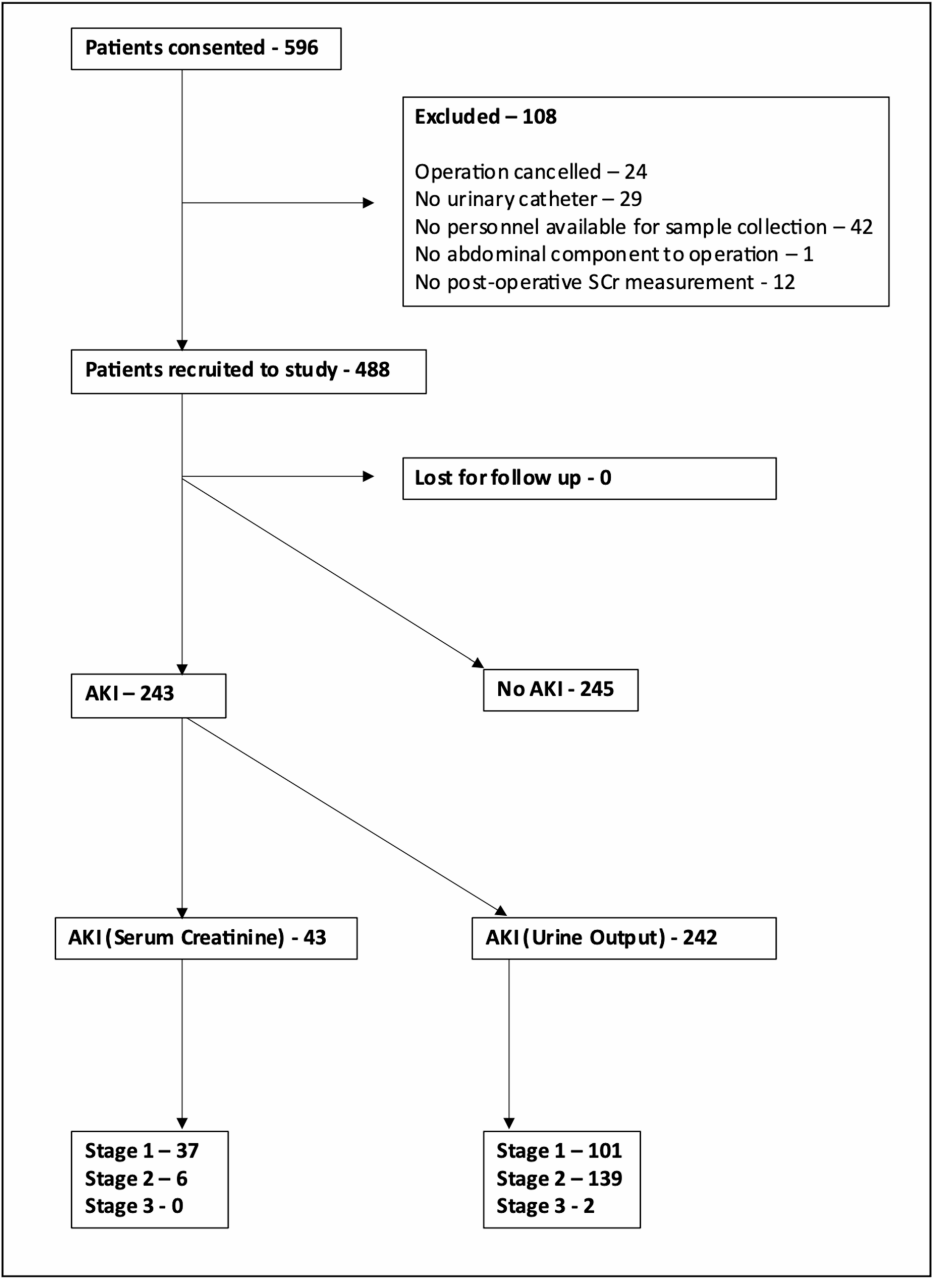
Consort diagram

**Development of AKI** : 243 (49.8%) of patients developed AKI of any stage using the KDIGO criteria. 37 patients (7.6%) developed stage 1 AKI-SCr, 6 patients (1.2%) stage 2, with no participants developing stage 3. 101 participants (20.7%) developed stage 1 AKI-UO and 139 (28.5%) stage 2 with two patients (0.4%) reaching stage 3 (Fig. 1). Of 51 emergency patients, seven (13.7%) developed AKI-SCr: six patients (11.8%) stage 1, and one patient stage 2 (1.9%). 30 (58.8%) patients developed AKI-UO, 10 (19.6%) stage 1 and 20 (39.2%) stage 2.

**Fig. 2:**
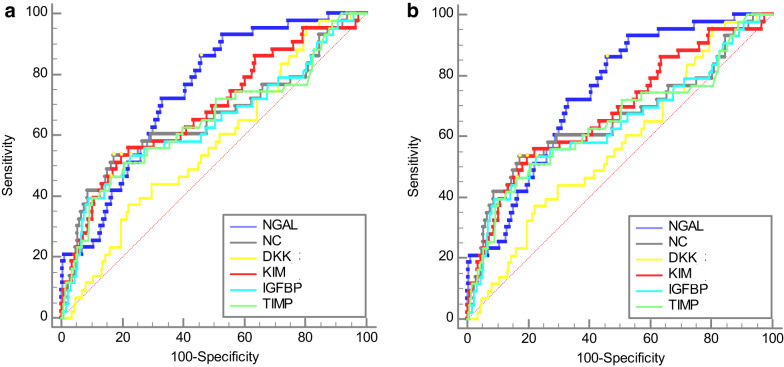
**a** ROC curves, all Biomarkers, AKI stages 1 and 2 by serum creatinine **b** ROC curves, all Biomarkers, AKI all stages by Urine Output

**Biomarker performance: **AUC values, sensitivity, specificity, and YI for each biomarker are shown in Tables 2 and 3. AUC values of NGAL for stage 1 AKI-SCr was 0.741 (95%CI 0.699–0.770, *p* < 0.001) and 0.871 (95%CI 0.838–0.899, *p* < 0.001) for stage 2. AUC values for NC for stage 1 were 0.655 (95% CI 0.611–0.697, p0.003) and stage 2 0.803 (95%CI 0.764–0.837 p0.002). The AUC for KIM-1 for stage 1 was 0.68 (95%CI 0.637–0.722, p.0.001); the AUC for stage 2 (0.743, 95%CI 0.702–0.781, p0.072) was not statistically significant. DKK-3 returned the lowest AUC values for all stages (Table 2; Fig. 2).

**Table 2:** Area Under the Receiver Operator Curve (AUROC) for prediction of AKI by SCr (Serum Creatinine) or UO (Urine Output) (Lower 95% CI.0.5 highlighted)

		**AUC for AKI prediction by SCr/UO stage**					
	**SCr all stages**	**SCr Stage 2**	**UO all stages**	**UO stage 2+**	**UO stage 3**	**SCr + UO all stages **	**SCr and UO, stage 2+**
**NGAL**	0.741	0.871	0.565	0.57	0.544	0.567	0.577
	(0.699–0.770)	(0.838–0.899)	(0.517–0.611)	(0.525–0.614)	(0.498–0.589)	(0.522–0.622)	(0.532–0.622)
	<0.001	<0.001	0.019	0.013	0.544	0.01	0.006
**NC**	0.655	0.803	0.587	0.625	0.825	0.585	0.626
	(0.611–0.697)	(0.764–0.837)	(0.539–0.633)	(0.579–0.670)	(0.787–0.860)	(0.540–0.629)	(0.581–0.669)
	0.003	0.002	0.001	<0.001	<0.001	0.001	<0.0001
**KIM-1**	0.68	0.743	0.587	0.614	0.567	0.585	0.616
	(0.637–0.722)	(0.702–0.781)	(0.540–0.633)	(0.567–0.659)	(0.522–0.612)	(0.540–0.629)	(0.571–0.660)
	<0.001	0.072	0.001	<0.001	0.657	0.001	0.0001
**DKK-3**	0.568	0.601	0.539	0.535	0.526	0.567	0.535
	(0.523–0.612)	(0.557–0.645)	(0.491–0.586)	(0.489–0.591)	(0.480–0.571)	(0.522–0.612)	(0.489–0.580)
	0.124	0.3576	0.158	0.221	0.952	0.0093	0.2179

NGAL (Neutrophil gelatinase-associated lipocalin), KIM-1 (Kidney injury molecule-1), DKK-3 (Dicckopf-related protein 3), IGFBP-7 (insulin-like growth factor binding protein-7) and TIMP-2 (tissue inhibitor of metaloproteinase-2)

**Table 3:** Diagnostic performance data at Y1 cut-off (AKI-SCr all stages)

	YI cut-off	Sensitivity	Specificity	PPV	NPV	PLR	NLR
**NGAL**	9.07	86.05	54.16	15.4	97.6	1.88	0.26
	(7.1–18.3)	(72.1–94.7)	(49.4–58.9)	(13.4–17.5)	(95.0–98.8)	(1.6–2.2)	(0.1–0.5)
**NC**	0.99	53.49	82.7	23	94.8	3.09	0.56
	(0.15–1.5)	(37.7–68.8)	(78.9–86.1)	(17.5–29.7)	(93.0–96.2)	(2.2–4.4)	(0.4–0.8)
**KIM-1**	2.5	53.49	80.45	20.2	94.5	2.74	0.58
	(0.74–3.2)	(37.7–68.8)	(76.5–84.0)	(15.2–26.4)	(92.6–95.9)	(2.0–3.8)	(0.4–0.8)
**DKK-3**	673.98	95.35	19.78	10.3	97.8	1.19	0.24
	(515.97–3071.1)	(84.2–99.4)	(16.2–23.8)	(9.6–11.1)	(91.8–99.4)	(1.1–1.3)	(0.06–0.9)

NGAL (Neutrophil gelatinase-associated lipocalin), KIM-1 (Kidney injury molecule-1), DKK-3 (Dicckopf-related protein 3), IGFBP-7 (insulin-like growth factor binding protein-7) and TIMP-2 (tissue inhibitor of metaloproteinase-2)

 All biomarkers had poor discrimination for stage 1 and 2 AKI-UO with AUCs of < 0.625. NC demonstrated an AUC of 0.83 (95%CI 0.787–0.860, *p* < 0.001) for stage 3 UO-SCr, with all other biomarkers returning values of < 0.6. Pairwise De Long tests comparing AUCs for prediction of AKI to NGAL to the other markers showed significant difference against DKK-3 (*p* = 0.003) but not against Nephrocheck (*p* = 0.1) or KIM-1 (*p* = 0.25).[37] In patients undergoing emergency surgery, AUC values for AKI-SCr and AKI-UO were statistically insignificant apart from NGAL at 0.808 (95% CI 0.674–0.905, *p* < 0.001). Median length of stay (LOS) was four days (IQR:3–7 days) for those who did not develop AKI. Those who developed AKI-SCr had a median LOS of twelve days (IQR 6–17 days), and those with AKI-UO of six days (IQR: 4–11 days). Two patients died within 30 days of surgery, ten within 90 days. Development of AKI-UO stage 2 or higher was associated with an increased risk of mortality within 90 days (RR 3.69, 95%CI: 1.05–12.88). 44 patients developed peroperative complications with a Clavien-dindo classification 3 or higher and was associated with the development of AKI postoperatively (RR 3.1163, 95% CI: 1.62–6.017). A breakdown of complications is provided in the supplement. No results for any biomarker were statistically significant for the prediction of 30 or 90-day mortality. Biomarker positive patients displayed a significantly longer median LOS. LOS was increased by 3 days if positive to NGAL (*p* < 0.001), 2 days for KIM-1 (*p* < 0.001) and NC (p0.001), and 1 day for DKK-3 (p.0.001). Biomarker positive results were associated with a significantly increased risk of developing AKI-SCr of any stage. NC and KIM-1 positivity was associated with an increased risk of developing AKIUO of any stage, and NGAL for stage 2 only (Table 4).

**Table 4:** Relative risk of developing aki/adverse outcome if biomarker positive

	AKI SCr all stages	AKI SCr stage 2	AKI UO all stages	AKI UO stage 2 +	AKI UO stage 3	Clavien 3+	90-day mortality
**NGAL**	6.2686	13.214	1.1605	1.3723	1.0165	2.2509	4.0661
	(2.6950–14.5809)	(0.7484–233.2972)	(0.9696–1.3889)	(1.0343–1.8208)	(0.06394–16.1604)	(1.2283–4.1247)	(0.8723–18.9539)
	**P** ** < 0.0001**	*P* = 0.0780	*P* = 0.1045	**P** **= 0.0283**	*P* = 0.9907	**P** **= 0.0087**	*P* = 0.0741
**NC**	4.462	7.76	1.2791	1.8187	3.88	1.5763	0.97
	(2.5552–7.7919)	(1.4418–41.7667)	(1.0558–1.5497)	(1.3779–2.4007)	(0.2448–61.4955)	(0.8599–2.8894)	(0.2092–4.4969)
	**P** ** < 0.0001**	**P = 0.0170**	**P = 0.0119**	**P** **< 0.0001**	*P* = 0.3362	*P* = 0.1411	*P* = 0.9690
**KIM-1**	3.9059	6.7928	1.4102	1.9246	0.2231	2.0621	2.2643
	(2.2297–6.8422)	(1.2607–36.5991)	(1.1800–1.6854)	(1.4684–2.5226)	(0.01409–3.5325)	(1.1729–3.6254)	(0.6504–7.8821)
	**P** **< 0.0001**	**P** **= 0.0258**	**P** **= 0.0002**	**P** **< 0.0001**	*P* = 0.2871	**P ** **= 0.0119**	*P* = 0.1991
**DKK-3**	4.5727	2.925	1.1264	1.2065	0.2231	3.1228	4.725
	(1.1269–18.5544)	(0.1663–51.4538)	(0.8777–1.4457)	(0.8148–1.7866)	(0.01409–3.5325)	(0.9902–9.8483)	(0.2794–79.8959)
	**P** **= 0.0334**	*P* = 0.4632	*P* = 0.3497	*P* = 0.3485	*P* = 0.2871	*P* = 0.0520	*P* = 0.2818

NGAL (Neutrophil gelatinase-associated lipocalin), KIM-1 (Kidney injury molecule-1), DKK-3 (Dicckopf-related protein 3), IGFBP-7 (insulin-like growth factor binding protein-7) and TIMP-2 (tissue inhibitor of metaloproteinase-2)

109 patients met the criteria for AKI Stage 1S using NGAL, 38 for NC, 40 for KIM-1 and 192 for DKK-3. 1 S patients for NGAL and DKK-3 had an LOS that was significantly longer than patients who were biomarker negative. The 1 S stage for NGAL was also associated with a higher risk of developing a significant complication. No other significant differences were seen in terms of adverse outcomes between the 1 S and biomarker negative non-AKI sub group (Table 5).

**Table 5:** Adverse outcomes, biomarker positive, non-AKI subgroup ‘1S’

	Cut-off	Positive	Negative	Median Length of Stay (IQR)			90-day mortality		Clavien 3+ Complication	
				Positive	Negative	*p*	Relative Risk if positive	*p*	Relative Risk if positive	*p*
**NGAL**	>9.07ug/l	109	136	5 (3–8)	4 (2–5)	**0.002**	2.4954 (0.233–27.159)	0.4528	5.6147 (1.238–25.450)	**0.0252**
**NC**	>0.98*	38	207	5 (3–6)	4 (3–7)	0.8071	0.7619 (0.041–14.462)	0.8561	1.2105 (0.272–5.385)	0.8025
**KIM-1**	>2.50ug/l	40	205	4 (3–7)	4 (3–7)	0.6241	0.7178 (0.038–13.635)	0.8253	1.1389 (0.256–5.076)	0.8645
**DKK-3**	>673.984ng/l	192	53	4 (3–7)	3.5 (3–7)	**0.0343**	1.9585 (0.103–37.334)	0.6549	6.4352 (3.854–107.462)	0.1949

NGAL (Neutrophil gelatinase-associated lipocalin), KIM-1 (Kidney injury molecule-1), DKK-3 (Dicckopf-related protein 3), IGFBP-7 (insulin-like growth factor binding protein-7) and TIMP-2 (tissue inhibitor of metaloproteinase-2)

### Long term outcomes

40 patients died within 1 year of their operation. Follow up data on renal function was obtained in 446 of 488 patients. Of the 42 patients who did not have follow up measurement of renal function, 15 died prior to 6 months and 27 were lost to follow up. Median time of follow up renal function measurement was 398 days post operatively (IQR: 366–490 days. 169 patients experienced an increase in CKD stage, 153 by one stage and 11 by two stages, 3 by three or more. No biomarker showed good predictive ability for a rise in CKD – all displayed AUC values of < 0.6. The relative risks of an increase in CKD stage if a patient was biomarker positive were also not significant. Similarly, AUC values for the prediction of mortality within one year were low. NGAL returned a statistically significant AUC (0.629, 0.585–0.672, p=0.0026) although all other AUC values for all other biomarkers were < 0.6. The association between NGAL positivity and 1 year mortality did not reach statistical significance (RR = 1.7742, 95% CI: 0.9111–3.4548, *p* = 0.0869). Full details are provided in Table 6.

**Table 6:** Long term follow up

**Mortality at 1 year**				
**Biomarker AUC, prediction of 1 year mortality**	**AUC**	**95% CI**	**p**	
NGAL	0.629	0.585 - 0.672	0.0026	
NC	0.506	0.460 - 0.551	0.8939	
KIM-1	0.56	0.515 - 0.605	0.1463	
DKK-3	0.526	0.481 - 0.571	0.5942	
**Relative risk of death at 1 year**	**RR**	**95% CI**	**p**	
No AKI	0.7132	0.3709 - 1.3712	0.3083	
AKI at all	1.4021	0.7293 - 2.6958	0.3108	
AKI Scr	2.4276	1.0030 - 5.8755	0.0492	
AKIUO at all	1.5826	0.8186 - 3.0594	0.1681	
AKIUO2 + 3	1.9313	0.9980 - 3.7373	0.0552	
NGAL positive	1.7742	0.9111 - 3.4548	0.0869	
NC positive	0.5308	0.2024 - 1.3920	0.168	
KIM-1 positive	0.9848	0.4540 - 2.1361	0.9691	
DKK-3 positive	1.2886	0.5239 - 3.1695	0.5713	
**Increase in CKD stage**		**n**		
Total		169		
Increase by 1		153		
Increase by 2		11		
Increase by 3		2		
Increase by 4		2		
Increase by 5		1		
**Biomarker AUC, prediction of increase in CKD stage at follow up**	**AUC**	**95% CI**	**p**	
NGAL	0.542	0.495 - 0.589	0.133	
NC	0.512	0.465 - 0.559	0.6739	
KIM-1	0.555	0.507 - 0.602	0.0527	
DKK-3	0.505	0.457 - 0.552	0.8746	
**Relative risk of increase in CKD stage if Biomarker positive**	**RR**	**95% CI**	**p**	
NGAL	1.3332	0.9085 - 1.9565	0.1412	
NC	0.9651	0.6030 - 1.5446	0.8822	
KIM-1	0.7321	0.4562 - 1.1749	0.1918	
DKK-3	0.6812	0.4232 - 1.0963	0.1155	
**Relative risk of increase in CKD stage if AKISCr/AKIUO**	**RR**	**95% CI**	**p**	
No AKI	0.8457	0.5766 - 1.2403	0.3908	
AKI at all	1.1825	0.8063 - 1.7342	0.3908	
AKISCr	1.7105	0.8482 - 3.4495	0.1354	
AKIUO	1.0152	0.6924 - 1.4885	0.9386	
AKIUO 2+3	1.3009	0.8534 - 1.9829	0.2226	
**Subgroup**				
Pts with normal renal function at baseline				365
Pts who developed CKD stage 3 at 1 year				12
**Relative Risk of developing CKD3+ depending on AKI stage**	**Yes**	**CKD3+**	**RR**	**p**
AKIUO at all	174	7	1.5368	0.4557
AKI UO2+	100	5	1.8929	0.266
AKISCr at all	22	3	5.197	0.0088
AKISCr 2+	6	1	5.4394	0.0777
**Biomarker performance, prediction of development of CKD3+ at 1 year**			**AUC**	**p**
NGAL			0.75	<0.001
NC			0.501	0.993
KIM-1			0.586	0.378
DKK-3			0.597	0.279

NGAL (Neutrophil gelatinase-associated lipocalin), KIM-1 (Kidney injury molecule-1), DKK-3 (Dicckopf-related protein 3), IGFBP-7 (insulin-like growth factor binding protein-7) and TIMP-2 (tissue inhibitor of metaloproteinase-2)

K-fold cross validation revealed a high degree of internal validity. Seven significant differences were seen among 210 different comparisons of demographic, outcome and modelling data between ‘test’ and ‘train’ subsets, suggesting homogeneity of the sample in line with the inclusion criteria. No significant differences were seen between the mean ‘test’ and study AUC values. Full K-fold cross validation results are available in the supplementary appendix.

## Discussion

To our knowledge, this is the largest reported investigation of biomarkers associated with AKI in patients undergoing major abdominal surgery. Biomarker performance was assessed in all patients and not just those deemed at high risk for AKI. Cases of AKI-SCr were predominantly mild (stage 1, 7.6%) with only six cases of stage 2 AKI (moderate, 1.2%) observed. As for AKI-UO, although the rate of both stage 1 and 2 were high (20.7% and 28.5% respectively), severe AKI was rare (two cases). No patients required renal replacement therapy. Our results differ from similar studies where higher rates of AKI were observed with over 14% as defined by SCr criteria and over 4% having stage 2 or 3 AKI [41]. Biomarker performance varies considerably depending on the AKI criteria chosen.

In individuals developing AKI-SCr, NGAL returned AUC values of 0.741 (95% CI: 0.699–0.770) for stage 1 and 0.871 (95% CI: 0.838–0.899) for stage 2. These figures are appreciably higher than for all other biomarkers studied and compares well with other widely used clinical biomarkers, such as troponin for myocardial injury [42]particularly with stage 2 AKI. Au et al. demonstrated an AUC of 0.74 (95%CI: 0.62–0.86) for NGAL in a mixed surgical population with a lower rate of AKI than this study although other research in major abdominal surgery has found lower AUC values [20, 43, 44]. Our AUC values determined for Nephrocheck are lower then those reported in cardiac surgery and the critically ill where AUCs of NC ranging from 0.68 to 0.85 for AKI of all stages are reported [23, 45]. Gocze et al. found an AUC for NC of 0.85 (95%CI: 0.78–0.93) for both stage 1 and 2 in a mixed high risk surgical cohort that included transplant and vascular procedures [23]. Our AUC values of 0.655 and 0.803 for stages 1 and 2 respectively are thus in keeping with other published data, but suggest that NC may not perform as well in comparatively low risk cohorts for AKI (non-cardiac or transplant surgery). KIM-1 performed moderately well in keeping with published data [46, 47]. DKK-3 was a poor predictor of AKI by either criteria (AUC of < 0.6), but there is only one study for comparison which assessed pre-operative DKK-3 levels in cardiac surgery (0.783, 95%CI 0.747–0.820) and as such its utility may be limited in the general post-operative setting [48].

No biomarker performed well for predicting AKIUO with AUC values < 0.65 for stages 1 and 2. Although NC demonstrated an AUC of 0.83 (95%CI 0.787–0.860, *p* < 0.001) for stage 3 AKI only two cases occurred. The poor performance of the biomarkers in predicting AKI-UO may reflect underlying transient oliguria whichis frequently observed in post-operative non-critically ill patients associated with vasopressin-release and sympathetic nervous system activation [41, 49]. We observed a rate of AKI-UO fivefold that of AKI-SCr implying that in this group the utility of UO-AKI may not be as useful reinforced by the finding that length of stay, complications and mortality are significantly higher in AKI-SCr than AKI-UO [50–52]. Likewise, we found a median length of stay with AKI-SCr double that of AKI-UO. It appears that classification AKI by UO or Cr do not equate to similar outcomes in post-abdominal surgical patients, and a reduction in UO post-operatively may reflect normal physiology. This is relevant when considering studies where AKI-UO and AKI-SCr have been assessed together [21, 22, 48]. No biomarker demonstrated good predictive ability for long-term outcomes including the development of CKD. This may reflect the relatively low event rate and suggests that biomarkers may be best employed in preventative strategies.

Our study has limitations. Despite best efforts, the sample was not truly consecutive given the unpredictable nature of operative time, personnel unavailability and clinical decisions omitting urinary catheters. K-fold cross validation does not however suggest any significant selection bias. Biomarkers were measured in laboratories using ELISA kits rather than point of care or hospital laboratory testing and differences in testing sites and methodology may have affected results, but both sets of laboratory technicians were blinded to participant data. Furthermore, biomarkers were collected at just one time point and future studies may address serial measurements which may improve prediction as well as highlighting patients at higher risk of AKI. However, strengths of the study include the prospective design, the inclusion of long term follow up as well as the use of multiple biomarkers reflecting different injury mechanisms. Also, to our knowledge this is the largest cohort study of biomarkers of AKI in general abdominal surgical patients.

## Conclusions

The utility of biomarkers associated with the development of AKI was found to be dependent on the method by which AKI was determined. In cases where AKI was defined solely on UO criteria performance was poor. In AKI-SCr NGAL was the best performing biomarker, with AUC values of both NGAL and NC improving with increasing AKI severity. NGAL compares well with other s biomarkers such as troponin in terms of diagnostic accuracy and future therapeutic studies are required to assess whether early AKI identification can translate into improved outcomes.

## Electronic supplementary material

Supplementary Material 1

## Data Availability

Research data supporting this publication are available from the University of Surrey Open Research repository: https://openresearch.surrey.ac.uk/esploro/outputs/doctoral/Urinary-biomarkers-for-the-prediction-of/99571623202346.
