# Supplementary material for: A prospective diagnostic study investigating urinary biomarkers of AKI in major abdominal surgery (the AKI-biomas study)
Source: Crit Care. 2025 Jul 1;29:260. doi: 10.1186/s13054-025-05510-8 (PMC12210991; doi:10.1186/s13054-025-05510-8)
Supplement: Supplementary file 1 — Supplementary Material 1 [file 13054_2025_5510_MOESM1_ESM.docx]

**Appendix 1. Collected Data Points**

1. Case Number

2. ID Number

3. Age

4. Sex

5. Height

6. Weight

7. ASA grade

8. Baseline Creatinine

9. Baseline ALP

10. Baseline Bilirubin

11. Baseline ALT

12. Baseline Bilirubin

13. Baseline ALT

14. Baseline Albumin

15. Baseline HB

16. Baseline Urea

17. Baseline WCC

18. Baseline eGFR

19. Diabetes 1 or 2

20. Hypertension yes/no

21. ACEI yes/no

22. ARB yes / no

23. Elective / Emergency

24. Operation date

25. Speciality

26. Operation

27. Approach

28. Blood Loss (<100mls, 100-499mls, 500-999mls, 1-2 litres, 2+ litres)

29. Intraoperative Inotrope duration

30. Intraoperative Fluid Administration

31. Intraoperative Blood product administration

32. Operation duration

33. Duration of Intraoperative Hypotension

34. Sample time

35. Urine Creatinine

36. Nephrocheck score

37. IGFBP-7

38. TIMP-2

39. NGAL

50. KIM-1

51. DKK-3

52. Post op Day 1/2/3 Creatinine

53. Post op Day 1/2/3 ALP

54. Post op Day 1/2/3 Bilirubin

55. Post op Day 1/2/3 Sodium

56. Post op Day 1/2/3 ALT

57. Post op Day 1/2/3 Albumin

58. Post op Day 1/2/3 Haemoglobin

59. Post op Day 1/2/3 Urea

60. Post op Day 1/2/3 Inotrope duration

61. Post op Day 1/2/3 Ventilation

62. Post op Day 1/2/3 Fluid Balance

63. Post op Day 1/2/3 Fluid administered

64. Post op Day 1/2/3 Insulin requirement

65. 1-12^th^ hour post-operative urine Output

66. AKI yes/no?

67. AKI urine output yes/no?

68. AKI urine output stage?

69. AKI serum creatinine yes/no?

70. AKI serum creatinine stage?

66. Complication yes/no

67. Complication Type (1-5)

68. Mortality (yes/no)

69. Date of death

70. Length of stay

71. Length of stay – ICU

72. Post-operative return to baseline renal function yes/no

73. Malignancy yes/no

**Appendix 2. Significant Complications**

| **Type** | **Detail** |  |  |  |  |  |  |  |  |  |
| --- | --- | --- | --- | --- | --- | --- | --- | --- | --- | --- |
| Clavien 3 | Requirement for pacemaker for bradycardia | | | | | | | | | |
| Clavien 3 | Blood transfusion, right lung chest drain for infection | | | | | | | | | |
| Clavien 3 | Gastroduodenal artery haemorrhage (re-admitted) and had embolisation | | | | | | | | | |
| Clavien 3 | Needed vacuum dressing for perineal wound | | | | | | | | | |
| Clavien 3 | Radiological drain to duodenal bed | | | | | | | | | |
| Clavien 3 | Pneumothorax, requiring chest drain | | | | | | | | | |
| Clavien 3 | Post op collections, CT guided drainage x3 | | | | | | | | | |
| Clavien 3 | CT guided drain pararectal collection. Problem with lithium / depression and refusal to eat post op. | | | | | | | | | |
| Clavien 3 | Required ERCP for bile leak, 2 units blood given in theatre | | | | | | | | | |
| Clavien 3 | Required exploration of jugular vein under GA, contrast leak into mediastinal drain | | | | | | | | | |
| Clavien 3 | Re-admitted with obstructed port site hernia and had open repair | | | | | | | | | |
| Clavien 3 | Pleural effusion, required chest drain | | | | | | | | | |
| Clavien 3 | Required US guided chest drain for anastomotic leak and oesophageal stent insertion | | | | | | | | | |
| Clavien 3 | Required dilatation of lower anastomotic stricture, 3 returns to ICU. Chest drains for effusions, tracheostomy | | | | | | | | | |
| Clavien 3 | Iatrogenic liver injury, liver abscess and pyloric stricture requiring dilatation | | | | | | | | | |
| Clavien 3 | CT guided drainage of presacral collection | | | | | | | | | |
| Clavien 3 | Liver collections, required ERCP | | | | | | | | | |
| Clavien 3 | Drainage of collection | | | | | | | | | |
| Clavien 3 | Pleural effusion requiring drain | | | | | | | | | |
| Clavien 3 | Return to theatre for further laparotomy and washout | | | | | | | | | |
| Clavien 3 | Return to theatre for further laparotomy and washout | | | | | | | | | |
| Clavien 3 | Drainage of collection | | | | | | | | | |
| Clavien 4 | Emergency re-operation for chest wall herniation, anastomotic leak, and endo sponge | | | | | | | | | |
| Clavien 3 | Emergency re-operation for leak | | | | | | | | | |
| Clavien 3 | Drainage of perisplenic collection | | | | | | | | | |
| Clavien 3 | Chest drain for pleural effusion | | | | | | | | | |
| Clavien 3 | Required transrectal drain of pelvic collection | | | | | | | | | |
| Clavien 3 | White out right lung requiring chest drain (biloma) | | | | | | | | | |
| Clavien 3 | Re-operation for sepsis - no cause found. | | | | | | | | | |
| Clavien 4 | Required embolisation, return to theatre (relook laparotomy) | | | | | | | | | |
| Clavien 5 | Death | | | | | | | | | |
| Clavien 4 | Required relook laparoscopy and washout | | | | | | | | | |
| Clavien 5 | Required gastroduodenal artery embolisation, death | | | | | | | | | |
| Clavien 4 | Had CPR, possibly secondary to ischaemic gut from incisional hernia (repaired) | | | | | | | | | |
| Clavien 4 | Required emergency oesophagectomy after abandoned procedure | | | | | | | | | |
| Clavien 4 | Hydropneumothorax requiring ITU | | | | | | | | | |
| Clavien 4 | Gluteal necrotising fascitiis requiring 2 debridements | | | | | | | | | |
| Clavien 5 | Death. large intrabdominal collection requiring drain | | | | | | | | | |
| Clavien 4 | Anastomotic leak on 7/7 requiring drain | | | | | | | | | |
| Clavien 4 | Return to theatre for colonic herniation | | | | | | | | | |
| Clavien 5 | Death | | | | | | | | | |
| Clavien 5 | Death | | | | | | | | | |
| Clavien 4 | Gastroduodenal artery bleed requiring embolisation and laparotomy | | | | | |  |  |  |  |
| Clavien 4 | Required re-operation and completion right hemicolectomy due to torted anastomosis | | | | | | | | | |

**Appendix 3. Risk prediction modelling**

**Stepwise risk prediction model without biomarkers**

The stepwise model revealed that age, ASA grade, BMI, Operation duration and Sex had a significant impact on the predictive ability of the model. Potentially included variables (save for sex) were tested for effect both as continuous (for example ‘68 years of age’) and categorical variables (for example ‘>over 50 years of age’). All variables had a more significant impact on the model as continuous variables.

Blood loss, CKD, Diabetes, Hypertension, Intraoperative fluid administration and gynae-oncological surgery were not included as they did not significantly impact the predictive ability of the model, despite being significantly associated with the development of AKI.

The AUC for the risk prediction model for predicting AKI was 0.795 (p=<0.001). This AUC is higher than all biomarkers included in the study. The Hosmer and Lemershow test also revealed good logistic regression model fit.

The logistic regression equation is:

$$Logit \left( p \right)=-11.71265+Age*0.051638+ASA*1.01147+BMI*0.078955+$$

$$Operation Duration*0.003515+Sex (Male-0.74993)$$

Regression coefficients for each variable

|  | **Coefficient** | **P** |
| --- | --- | --- |
| **Age** | 0.051638 | 0.0028 |
| **ASA** | 1.01147 | 0.0005 |
| **BMI** | 0.078955 | 0.003 |
| **Operation duration** | 0.003515 | 0.0015 |
| **Sex (Male)** | 0.74993 | 0.0378 |
| **Constant** | -11.71265 | <0.0001 |

**Biomarkers in conjunction with the stepwise risk prediction model**

Apart from DKK-3, all biomarkers were found to have a significant impact on the predictive ability of the stepwise model and were thus included. They all increased the AUC value. The same five variables from the initial stepwise models remained the same. NGAL was found to have the most significant impact on the model and was thus preferentially included over all other biomarkers by the statistical program. When assessing the difference between the model and biomarker-models, TIMP-2 was found to return the highest AUC, a difference that was statistically significant. KIMNC and NC were also shown to make a statistically significant difference.

**AUC values and differences with stepwise model**

|  | **AUC** | **DBA** | **95% CI** | **Significance** | **%** |
| --- | --- | --- | --- | --- | --- |
| **Stepwise** | 0.795 | - | - | - | 91.6 |
| **NGAL** | 0.821 | 0.0256 | -0.03 – 0.04 | 0.1392 | 92.01 |
| **NC** | 0.815 | 0.02 | **0.01 – 0.04** | **0.0128** | 91.8 |
| **KIM-1** | 0.815 | 0.00198 | -0.02 – 0.02 | 0.138 | 92.42 |
| **IGFBP-7** | 0.824 | 0.0287 | -0.01 – 0.06 | 0.0631 | 90.78 |
| **TIMP-2** | 0.825 | 0.0292 | **0.01 – 0.06** | **0.0322** | 91.8 |
| **NGALKIM** | 0.82 | 0.0246 | -0.01 – 0.06 | 0.1392 | 91.39 |
| **NGALNC** | 0.819 | 0.0241 | -0.01 – 0.06 | 0.149 | 92.21 |
| **KIMNC** | 0.809 | 0.137 | **0.01 – 0.03** | **0.0224** | 91.6 |

***DBA -*** *DBA – difference in AUC with stepwise model, % - % of cases correctly classified*

Regression coefficients for each variable and best performing biomarker (NGAL)

|  | **Coefficient** | **P** |
| --- | --- | --- |
| **Age** | 0.053184 | 0.0035 |
| **ASA** | 0.89519 | 0.0035 |
| **BMI** | 0.09121 | 0.0009 |
| **Operation duration** | 0.0029796 | 0.0109 |
| **Sex (Male)** | 0.80987 | 0.0323 |
| **NGAL** | 0.0015914 | 0.0003 |
| **Constant** | -12.00817 | <0.0001 |

The logistic regression equation is:

$$Logit \left( p \right)=-12.008+Age*0.053+ASA*0.895+ BMI*0.091+$$

$$Operation Duration*0.002+NGAL*0.0016+Sex (Male-0.809)$$

**Appendix 4. K-Fold Cross Validation**

Table 1 is scaled down and colour coded to show whether each participant was in the ‘test’ or ‘train’ group as part of k-fold validation. Each participant was in the ‘train’ cohort 4 times, and ‘test’ cohort 1 time (K value = 5, red = train, white = test).

Table 1. Allocation of participants to test and train cohort, K-fold Cross Validation

42 different demographic, operative, biomarker, model and outcome parameters were compared. Across 210 comparisons in the 5 cross validation folds, 7 significant differences were seen between ‘test’ and ‘train’ cohorts. These are shown in the following table 2.

Table 2. Differences between ‘Test’ and ‘Train’ Cohorts, K-Fold Cross Validation

**Mann-Whitney test, **Chi-squared test*

With regards to the AUC of the biomarkers at predicting AKI by serum creatinine, six significant differences were seen between test and train cohorts out of 45 comparisons. The low number of significant differences between test and train cohorts across the K-fold cross validation process reveals a high degree of homogeneity within the study population.

Table 3. Differences between AUC values, Test and Train Cohorts, K-Fold Cross Validation

**Mann-Whitney test*

Mean ‘Test’ AUC and study AUC values are provided in table 4. No significant differences were seen between mean AUCs from cross validation and the entire study cohort.

Table 4. Mean Test AUC values and Study AUC values

|  | **Mean 'Test' AUC** | **Study AUC** |
| --- | --- | --- |
| **NGAL** | 0.736 | 0.741 |
| **NC** | 0.664 | 0.655 |
| **KIM-1** | 0.693 | 0.680 |
| **DKK-3** | 0.572 | 0.568 |
| **IGFBP** | 0.674 | 0.639 |
| **TIMP** | 0.657 | 0.647 |
| **NGALKIM** | 0.767 | 0.767 |
| **NGALNC** | 0.734 | 0.739 |
| **KIMNC** | 0.687 | 0.679 |

**Appendix 5. Biomarker values by AKI stage**

|  |  | **Biomarker values by AKI stage** | | | | | | |
| --- | --- | --- | --- | --- | --- | --- | --- | --- |
|  |  | **No AKIScr** | **AKISCr stage 1** | **AKISCr stage 2** | **NoAKIUO** | **AKIUO stage 1** | **AKIUO stage 2** | **AKI UO stage 3** |
| **NGAL** | **Min** | 0.23 | 2.36 | 20.01 | 0.26 | 0.23 | 0.45 | 4.48 |
|  | **Max** | 2451 | 2620 | 3216 | 1078.7 | 3216 | 2620 | 16.03 |
|  | **Median** | 8.03 | 25.41 | 383.52 | 7.56 | 8.56 | 13.88 | 10.26 |
|  | **IQR** | 3.81-27.54 | 10.12-65.86 | 20.78-2183.00 | 3.44-27.86 | 4.12-30.01 | 5.22-38.64 | 4.48-16.03 |
| **NC** | **Min** | 0.01 | 0.05 | 0.26 | 0.01 | 0.01 | 0.02 | 0.98 |
|  | **Max** | 18.61 | 7.33 | 3.3 | 18.61 | 4.1 | 9.46 | 1.39 |
|  | **Median** | 0.42 | 0.99 | 1.91 | 0.394 | 0.39 | 0.61 | 1.19 |
|  | **IQR** | 0.20-0.81 | 0.18-1.91 | 0.69-3.16 | 0.18-0.69 | 0.23-0.74 | 0.26-1.22 | 0.98-1.39 |
| **KIM-1** | **Min** | 0.01 | 0.17 | 0.61 | 0.03 | 0.01 | 0.09 | 1.06 |
|  | **Max** | 11.59 | 11.07 | 13.72 | 11.07 | 6.46 | 13.72 | 2.09 |
|  | **Median** | 1.28 | 2.51 | 3.814 | 1.19 | 1.37 | 1.77 | 1.58 |
|  | **IQR** | 0.70-2.10 | 0.99-4.09 | 1.29-8.11 | 0.67-1.92 | 0.71-2.12 | 0.81-3.19 | 1.07-2.09 |
| **DKK-3** | **Min** | 37.45 | 228.99 | 804.01 | 37.45 | 73.54 | 127.24 | 394.3 |
|  | **Max** | 195971.2 | 40054.5 | 7674.38 | 195971 | 113107 | 71778.5 | 29803 |
|  | **Median** | 1609.42 | 1825.47 | 3424.59 | 1523 | 1786.4 | 1711.73 | 15099 |
|  | **IQR** | 797.38-3858.51 | 1020.34-5612.95 | 941.39-5212.03 | 745.80-3496.91 | 1039.37-5163.89 | 959.71-4727.06 | 394.30-29803.24 |
